# The emergence of the cause of rare diseases and rare disease patients’ movement

**DOI:** 10.1186/1750-1172-7-S2-A32

**Published:** 2012-11-22

**Authors:** Caroline Huyard

**Affiliations:** 1 Ceraps, Université Lille 2, Lille, France

## 

Three features of the cause of rare diseases may be important for the future of the movement.

1. The category of rare diseases was originally created to remedy the problem caused by orphan drugs in the USA. The 1962 Kefauver-Harris amendments to the Food, Drug and Cosmetic Act required the company wanting to market a drug to prove its efficacy. Each drug had to be (retroactively) tested. Drugs that had not been tested however remained in hospital pharmacies, and were called "homeless" or "orphan". Meanwhile, some U.S. patients were forced to discontinue their treatment. In the 1970s, these two phenomena pushed the FDA to try and find a status for the drugs that, although they had been developed, were not available for the patients. Defining these drugs as drugs for rare diseases appeared as the solution. This was the beginning of the rare diseases movement.

2. Facing a rare disease, the patients and their families often feel isolated. A series of interviews with 29 French patients and 15 relatives who were affected by one of 6 rare disorders suggests that, once illness has become an important aspect in the life of a person with a rare disease, or a of person related to one with a rare disease, this person will try to meet other people in the same situation.

3. Rare disease organizations are issue-based (people gather to struggle with common problems that they want to solve together) while coalitions of such organisations tend to be rarity-based (organisations come together because their members address rare disorders). A series of 61 interviews with patients, relatives, volunteers and employees in 8 French associations identified three solidarity principles for these coalitions: (1) the "one for all" principle, where any advancement for one disorder will benefit many others; (2) the "every man for himself" principle, where one should support their own rare disorder organisation; (3) the "all for one" principle, where solidarity is moral. None of these principles is satisfying. Gathering people with/or related to rare disorders with others addressing the same issues, i.e. an issue-based approach in ad-hoc coalitions, has the potential to be strong and cohesive. E.g.: coalitions of rare disease associations with Aids or cancer associations for access to insurance or credit.

